# P-1160. Hospital-Onset Bacteremia as a Surveillance Tool: A Comparative Analysis with CLABSI Rates

**DOI:** 10.1093/ofid/ofaf695.1353

**Published:** 2026-01-11

**Authors:** Xaviera Ortiz Soto, Jose Martinez, Kelly R Reveles, Lauren N Garza, Jose Cadena

**Affiliations:** University of Texas Health Science Center at San Antonio, San Antonio, TX; South Texas Veterans Health Care System, San Antonio, Texas; The University of Texas at Austin, Austin, Texas; South Texas Veteran Health Care System, San Antonio, Texas; South Texas Veterans Health Care System, UT Health San Antonio, San Antonio, Texas

## Abstract

**Background:**

Hospital-acquired infections (HAIs) are the most frequent cause of nosocomial adverse effects in the United States. In 2009, the Department of Health and Human Services developed an action plan to eliminate HAIs which includes monitoring the rates of six nosocomial infections including central line-associated bloodstream infections (CLABSIs). The focus on these measures has resulted in a decreased incidence of nosocomial blood stream infections, but the measures are labor-intensive, subject to ceiling effects, and do not reflect the full burden of hospital-acquired blood stream infections. Given these limitations, hospital-onset blood stream infection (HOBSI), defined as the growth of a recognized bacterial or fungal pathogen from a blood culture specimen collected on or after the fourth day of hospital admission, has been proposed as an alternate measure of nosocomial blood-stream infections. In a prior study, HOBSI rate correlated with CLABSI rate making this a possible surrogate marker for CLABSI.

**Figure:**
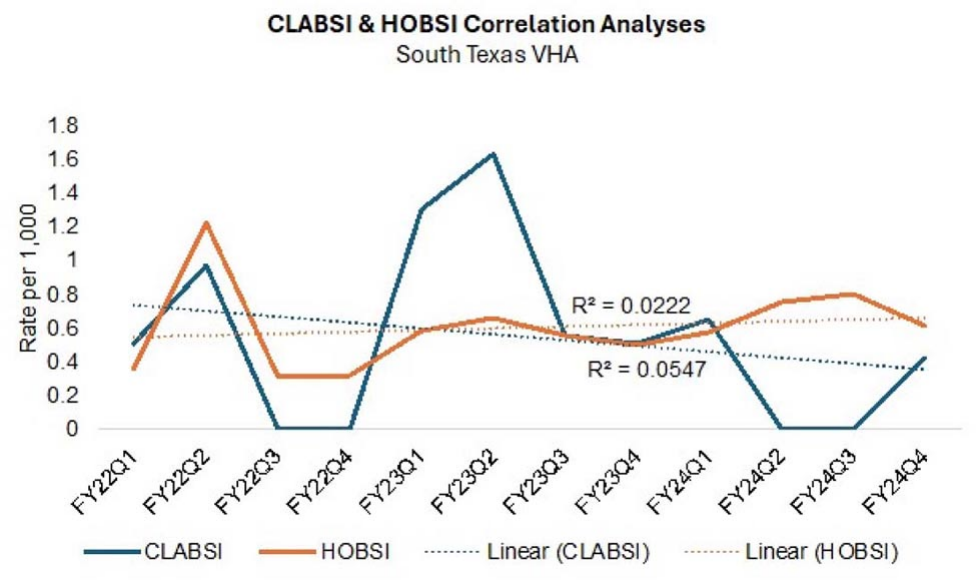
CLABSI & HOBSI Correlation Analyses South Texas VHA

**Methods:**

This was a prospective review of all blood cultures positive for non-commensal organisms on day 4 or later of admission for patients hospitalized at the Audie L. Murphy VA between January 1, 2022 and December 31, 2024. Trends in HOBSI rates per 1000 bed days of care and CLABSI rates per 1000 central line days were described quarterly throughout the study period. The relationship between HOBSI and CLABSI was evaluated using Pearson correlation.

**Results:**

There were 91 total HOBSI and 14 CLABSI during the period reviewed. There was a trend towards an increase in HOBSI rates and decreasing CLABSI rates, with significant variability over time resulting in low R2 values.

There was a low, non-significant correlation between CLABSI and HOBSI rates (R=0.30; p=0.343).

**Conclusion:**

HOBSI surveillance has been proposed as an alternate nosocomial blood-stream surveillance measure that would be easier to automate and would provide data on hospital-acquired blood stream infections beyond CLABSIs. In our study, there was not a significant correlation between HOBSI and CLABSI rates, but rates of both were low. Further research is needed into whether HOBSI surveillance should be adopted as part of our standard HAI surveillance measures.

**Disclosures:**

Kelly R. Reveles, PharmD, PhD, AstraZeneca: Advisor/Consultant|Ferring Pharmaceuticals: Advisor/Consultant

